# Explaining among-country variation in COVID-19 case fatality rate

**DOI:** 10.1038/s41598-020-75848-2

**Published:** 2020-11-03

**Authors:** Gabriele Sorci, Bruno Faivre, Serge Morand

**Affiliations:** 1 Biogéosciences, CNRS UMR 6282, Université de Bourgogne Franche-Comté, 6 Boulevard Gabriel, 21000 Dijon, France; 2 CNRS ISEM - CIRAD ASTRE - Montpellier Université, Montpellier, France; 3 Faculty of Tropical Medicine, Mahidol University, Bangkok, Thailand

**Keywords:** Pathogens, Viral infection

## Abstract

While the epidemic of SARS-CoV-2 has spread worldwide, there is much concern over the mortality rate that the infection induces. Available data suggest that COVID-19 case fatality rate had varied temporally (as the epidemic has progressed) and spatially (among countries). Here, we attempted to identify key factors possibly explaining the variability in case fatality rate across countries. We used data on the temporal trajectory of case fatality rate provided by the European Center for Disease Prevention and Control, and country-specific data on different metrics describing the incidence of known comorbidity factors associated with an increased risk of COVID-19 mortality at the individual level. We also compiled data on demography, economy and political regimes for each country. We found that temporal trajectories of case fatality rate greatly vary among countries. We found several factors associated with temporal changes in case fatality rate both among variables describing comorbidity risk and demographic, economic and political variables. In particular, countries with the highest values of DALYs lost to cardiovascular, cancer and chronic respiratory diseases had the highest values of COVID-19 CFR. CFR was also positively associated with the death rate due to smoking in people over 70 years. Interestingly, CFR was negatively associated with share of death due to lower respiratory infections. Among the demographic, economic and political variables, CFR was positively associated with share of the population over 70, GDP per capita, and level of democracy, while it was negatively associated with number of hospital beds ×1000. Overall, these results emphasize the role of comorbidity and socio-economic factors as possible drivers of COVID-19 case fatality rate at the population level.

## Introduction

At the end of 2019, a novel coronavirus (SARS-CoV-2) emerged from an animal reservoir in the city of Wuhan, China^1,2^. Having established a human-to-human transmission, the virus rapidly spread, first within China, and subsequently outside China, worldwide. On 11th March 2020 the WHO declared SARS-CoV-2 as a pandemic^3^. The coronavirus disease 2019 (COVID-19) produces a series of respiratory symptoms that vary in severity^4–10^. Although in most people the infection produces an asymptomatic disease or mild symptoms that do not require particular medical care, in a fraction of patients the disease develops into severe respiratory distress, due to an overreacting inflammatory response, requiring hospitalization in intensive care units. In the absence of specific treatments that prevent or block viral replication, a serious matter of concern is the proportion of infected patients that will eventually die. Current data indicate that, worldwide, case fatality rate (CFR, the ratio between number of deaths and number of confirmed cases) might be around 4%. However, at the country level, CFR ranges from 0 to more than 20%. There are many possible reasons for such a variation^11^. First, the epidemic has spread in some countries earlier than in others and therefore the difference in CFR might reflect different stages during the spread of the disease. According to this hypothesis, one might expect an increase in CFR with time in countries where CFR is currently low. Second, at the individual level, clinical data have reported several risk factors associated with a poor prognosis. Age and comorbidities (cardiovascular diseases, cancers, diabetes mellitus, chronic lung diseases) seem to greatly increase the mortality risk^12–15^. Therefore, countries with a greater share of elderly in the population, or with higher incidence of recognized comorbidity factors might pay the highest toll to the infection. Third, as said before, in the absence of effective treatments, patients that develop severe symptoms require hospitalization in intensive care units for respiratory assistance. A major concern is that, as the number of infected people increases, the healthcare system will be overwhelmed^16^. Under this scenario, mortality rate might reflect the country-specific capacity to tackle a large number of patients requiring respiratory assistance and intensive care. Finally, given that CFR is defined as the ratio between number of deaths and number of confirmed cases, countries might simply differ in the accuracy with which they detect the infection. Indeed, CFR estimates are prone to error if the actual number of infected people is much higher that the number of PCR confirmed cases (producing an overestimated CFR), or if mortality occurs with a delay (producing an underestimated CFR). Therefore, variation in CRF might reflect among country variation (i) in population screening (i.e., the number of tests performed), which affects the denominator of the ratio between number of deaths and number of confirmed cases, (ii) in counting and communicating the actual number of patients that have succumbed from SARS-CoV-2 infection.

Here, we conducted an analysis of the factors that might account for the variability of COVID-19 CFR among countries. Using data updated to June 11th 2020, we first investigated if CFR significantly varies between countries, independently from (i) the stage of the epidemic wave, (ii) the testing strategy, and (iii) the social distancing policies adopted by each country. Second, we used country-specific variables assessing the occurrence of comorbidities, as well as demographic, economic and political variables to uncover any association pattern with COVID-19 CFR.

## Methods

We used data on daily number of confirmed cases and deaths for each country reported by the European Center for Disease Prevention and Control (ECDC). We computed the case fatality rate as the ratio between deaths and confirmed cases. We restricted the dataset to countries with at least 100 confirmed cases to avoid spurious results due to small numbers. For each country, we also counted the number of days between the 100th case and 11th June 2020, and the number of days from the occurrence of at least one death ×1,000,000, indicating the progression of the epidemic.

We used the online resource https://ourworldindata.org/ to retrieve data on the incidence of known comorbidity factors for each country, as well as information on demographics, economics and political regimes. In particular, we used different metrics to describe comorbidities: (1) disability-adjusted life years (DALYs); (2) share of total disease burden; (3) age-standardized death rates per 100,000; (4) share of deaths. We focused on known comorbidities such as cardiovascular diseases, cancers, chronic respiratory diseases, diabetes mellitus, chronic kidney diseases. We also included metrics related to factors that might impinge on the severity of respiratory syndromes, such as smoking and air pollution. We also used https://ourworldindata.org/ to retrieve information on demographic, economic and political indicators. Table S1, in the supplementary information, reports the list, description and source of the variables used here, according to the GATHER statement^17^.

### Statistical analyses

We first aimed at exploring whether CFR varied among countries while controlling for the differences in the epidemic progression. To this purpose, we run a linear mixed model (LMM) where CFR was the dependent variable, time since 100th case (in days) and squared time since 100th case (as to model non-linear variation), country and the two-way interactions were the fixed factors. Country was also included as a random effect. For computational reasons, in this model the covariance structure of the R matrix was modeled using variance components, and degrees of freedom were computed using the between-within method. Only countries for which at least 10 days had elapsed between the record of the 100th case and 11th June 2020, and for which number of deaths was higher than 1 per 1,000,000 inhabitants were included in this model, to allow a better estimate of the variation between CFR and time. This model included 143 countries and 8441 daily values of CFR. In a second model, we also included the number of tests performed ×1000 as to control for differences in testing strategies among countries, and a stringency index describing the severity of the social distancing rules adopted by each country. This reduced the number of countries included in the model to 72 and the number of total observations to 3778.

To assess the pattern of association between country-specific CFR and comorbidities, we ran four LMMs that included different metrics as fixed factors. The first model included DALYs lost to cardiovascular, cancer and chronic respiratory diseases (due to the strong correlations among variables, we summed the three DALYs and used the sum in the model), and DALYs lost ×100,000 for people older than 70 years. The second model included share of disease burden (due to cardiovascular, cancer and chronic respiratory diseases). The third model included age-standardized death rates (due to cardiovascular diseases, cancer, air pollution, ambient particulate matter pollution, and smoking for people older than 70 years). The last model included share of deaths (due to cardiovascular, cancer, chronic respiratory, kidney diseases, lower respiratory infections, diabetes mellitus, outdoor air pollution). The four models always included a set of covariates that described demographic, economic and political variables, namely population size, share of the population over 70 years, gross domestic product (GDP) per capita, total health care expenditure as share of GDP, number of hospital beds (×1000 inhabitants), political regime, stringency index, and the number of tests performed ×1000. Political regime is scored according to the level of democracy, between − 10 (full autocracy) to + 10 (full democracy) (Polity IV as reported in https://ourworldindata.org/). The stringency index describes the severity of the policies implemented by each country to limit the spread of the virus, according to the following categories: school closure, workplace closure, public events cancelled, restrictions on gatherings, public transport closure, public information campaigns, stay at home, restriction on internal movements, international travel controls, testing policy, contact tracing. Finally, all models also included time since 1 death ×1,000,000 was reached (in days), and squared time as to take into account the progression of the epidemic in each country. In each model, we tested the interaction between the different comorbidity, demographic, economic factors and time. This allowed us to ascertain whether the temporal changes in CFR differed as a function of country-specific comorbidity, demographic, and socio-economic factors. All variables were standardized with mean = 0 and standard deviation = 1 to make parameter estimates directly comparable (variables with asymmetrical distribution were previously log-transformed to reduce skewness). To take into account the covariation between observations at different geographical scale, the LMMs also included three nested effects as random variables: continent, geographic region^18^ nested within continent, country nested within geographic region nested within continent. The covariance structure of the R matrix was modeled using a first-order autoregressive structure to model the temporal autocorrelation between daily CFR values. Degrees of freedom were computed using the Satterthwaite approximation. In order to minimize the risk of false discovery rate, we set the value of α to 0.01. Therefore, only p values lower than 0.01 were considered as indicative of statistically significant associations.

All the analyses were conducted with SAS 14.3 (PROC MIXED).

## Results

**Figure 1:**
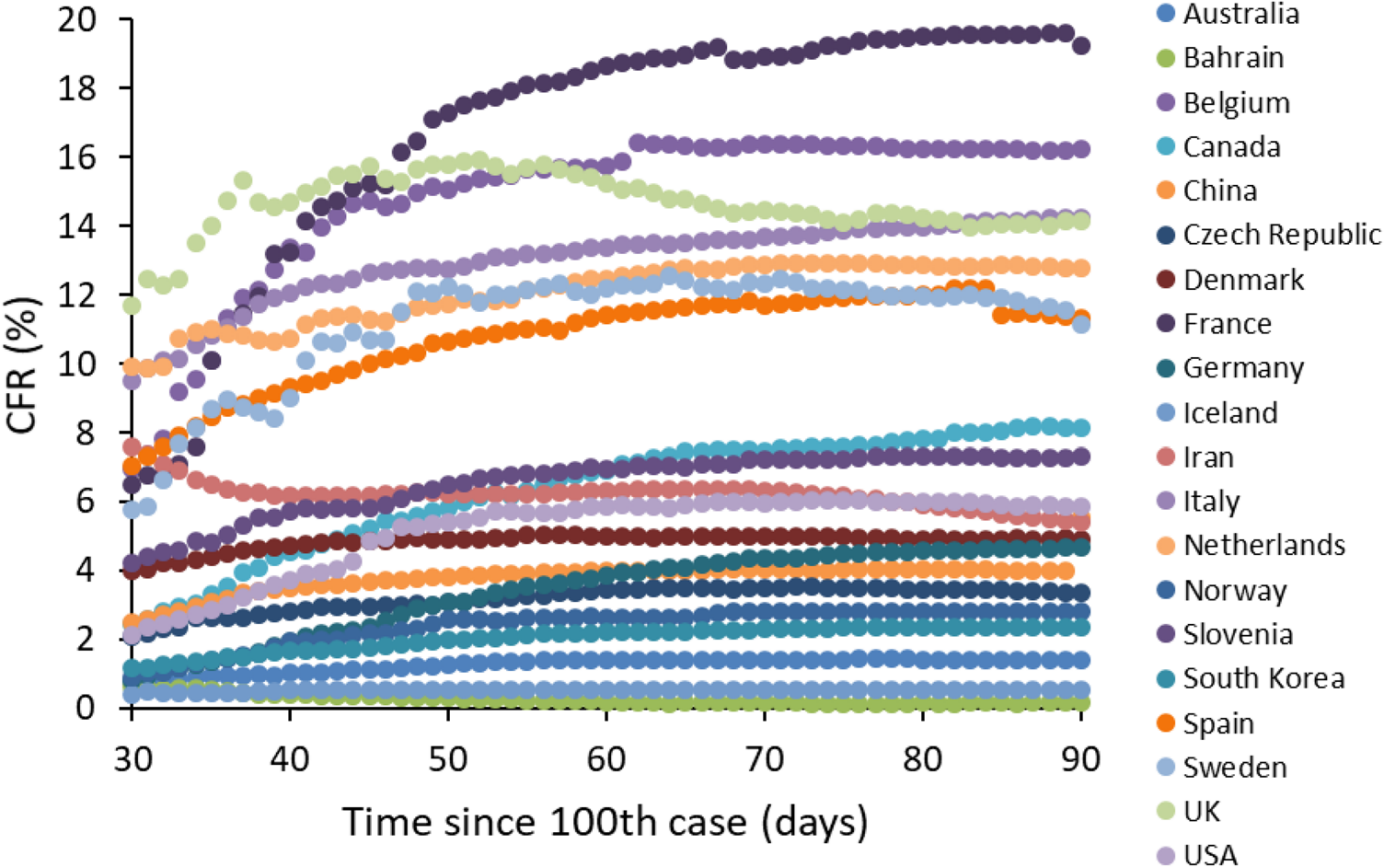
Time-dependent variation in COVID-19 case fatality rate (CFR) among countries. Time refers to the period between 30 and 90 days post 100th case. For illustrative reasons, only 20 countries are reported here.

**Figure 2:**
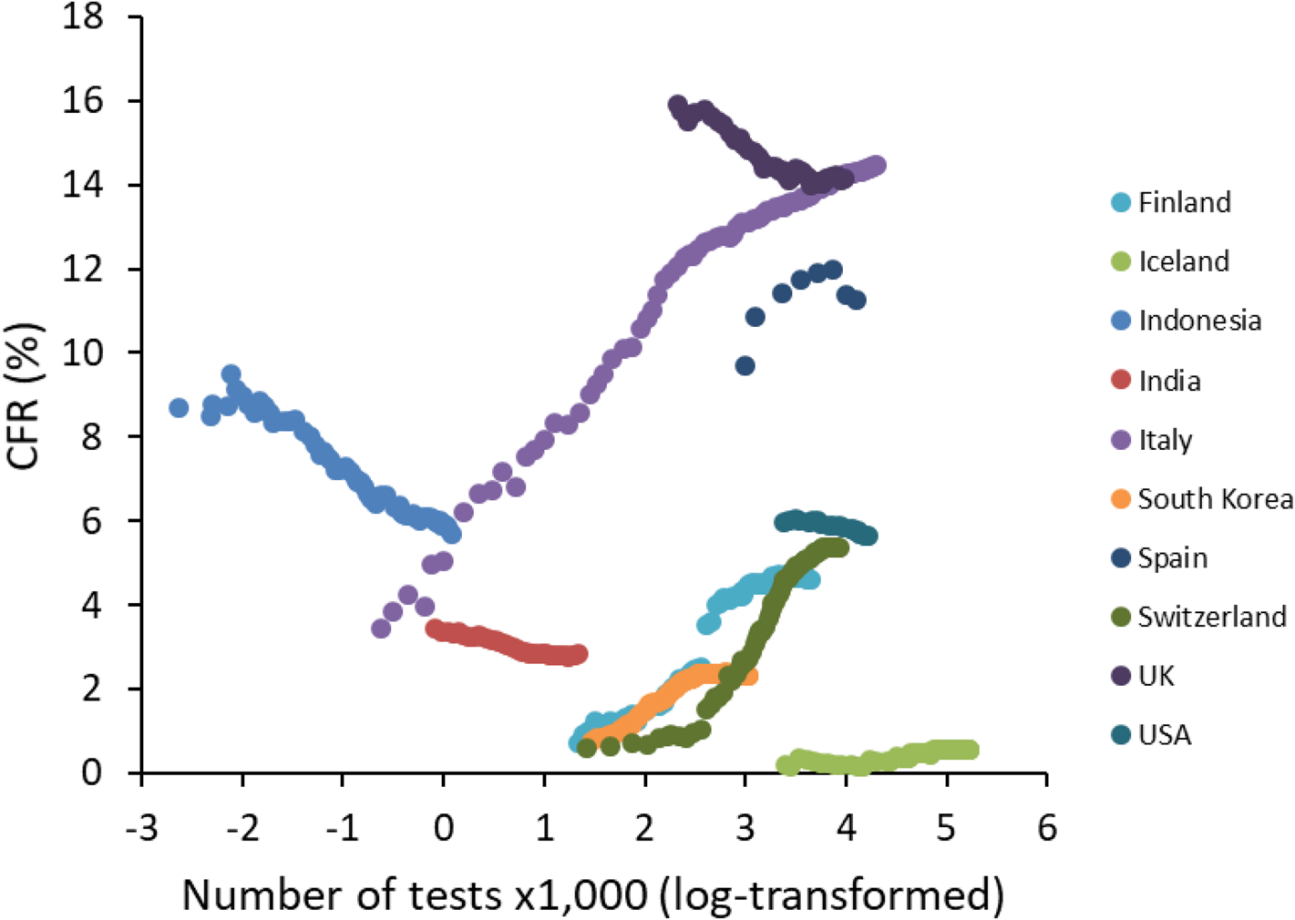
Changes in COVID-19 CFR as a function of the number of tests performed (×1000). For illustrative reasons, we report some representative countries showing how the relationship between CFR and number of tests can vary from negative to positive.

We found strong evidence for among-country differences in COVID-19 CFR. The LMM showed a highly significant interaction between time since the 100th case and country, indicating that CFR trajectories (both linear and quadratic components), did differ among countries as the epidemic progressed (Table 1, Fig. 1). CFR is defined as the ratio between number of deaths and number of confirmed cases. Since many cases of asymptomatic infection (or infections with mild symptoms) might get unnoticed, CFR is certainly an overestimate of the actual risk of dying when infected with SARS-Cov-2. In the light of this argument, a strategy of massive screening of the population (not only patients who are admitted to the hospitals, but also those with no or mild symptoms) might provide a more realistic estimate of the denominator of the CFR. Although the number of tests performed over the course of the epidemic is available for a smaller number of countries, it nevertheless varies tremendously among them, indicating different screening strategies adopted by each country. Focusing, for instance, on day 80 since the 100th case, and including only countries with more than 1 death per 1,000,000 inhabitants (n = 50 countries), number of tests performed per 1000 inhabitants varied from 0.9 (Indonesia) to 179 (Iceland). Interestingly, however, countries that better screened their population did not always suffer from the lowest CFR. At day 80 since the 100th case, there was no correlation between number of tests per 1000 inhabitants and CFR (Pearson’s r = − 0.101, *p* = 0.4873, n = 50). Using the whole dataset, and including the number of tests ×1000, showed that the relationship between CFR and number of tests was country specific, with some countries where screening resulted in a decline in CFR, whereas in other the relationship was positive (interaction between country and number of tests ×1000: F_71,3782_ = 23.17, *p* < 0.0001; Fig. 2). Importantly, the interaction between time and country remained highly significant even when adding both the number of tests ×1000 and the stringency index in the model (time × country: F_69,3429_ = 43.50, *p* < 0.0001; squared time × country: F_69,3429_ = 45.88, *p* < 0.0001). This result, therefore, suggests that variation in CFR among countries does not merely depend on the screening effort provided by each country, nor on the severity of social distancing and isolation rules.

**Table 1:** Linear mixed model exploring variation of COVID-19 case fatality rate (CFR) as a function of time since the 100th case for each country.

Fixed effects	F	*df*	*p*
Country	2484.95	142, 8012	** < 0.0001**
Time since 100th case (days)	0.10	1, 8012	0.7518
Squared time since 100th case	0.01	1, 8012	0.9293
Country × time since 100th case	193.48	142, 8012	** < 0.0001**
Country × squared time since 100th case	109.93	142, 8012	** < 0.0001**

The model also included squared time since 100th case and the interactions between country and time. Country was also declared as a random effect in the model. The model was restricted to countries that had 10 or more days elapsed between the occurrence of the 100th case and 11th June 2020 and for which number of deaths was higher than 1 per 1,000,000 inhabitants. The analysis is based on 143 countries and 8441 observations. Significant p-values are in bold.

**Figure 3:**
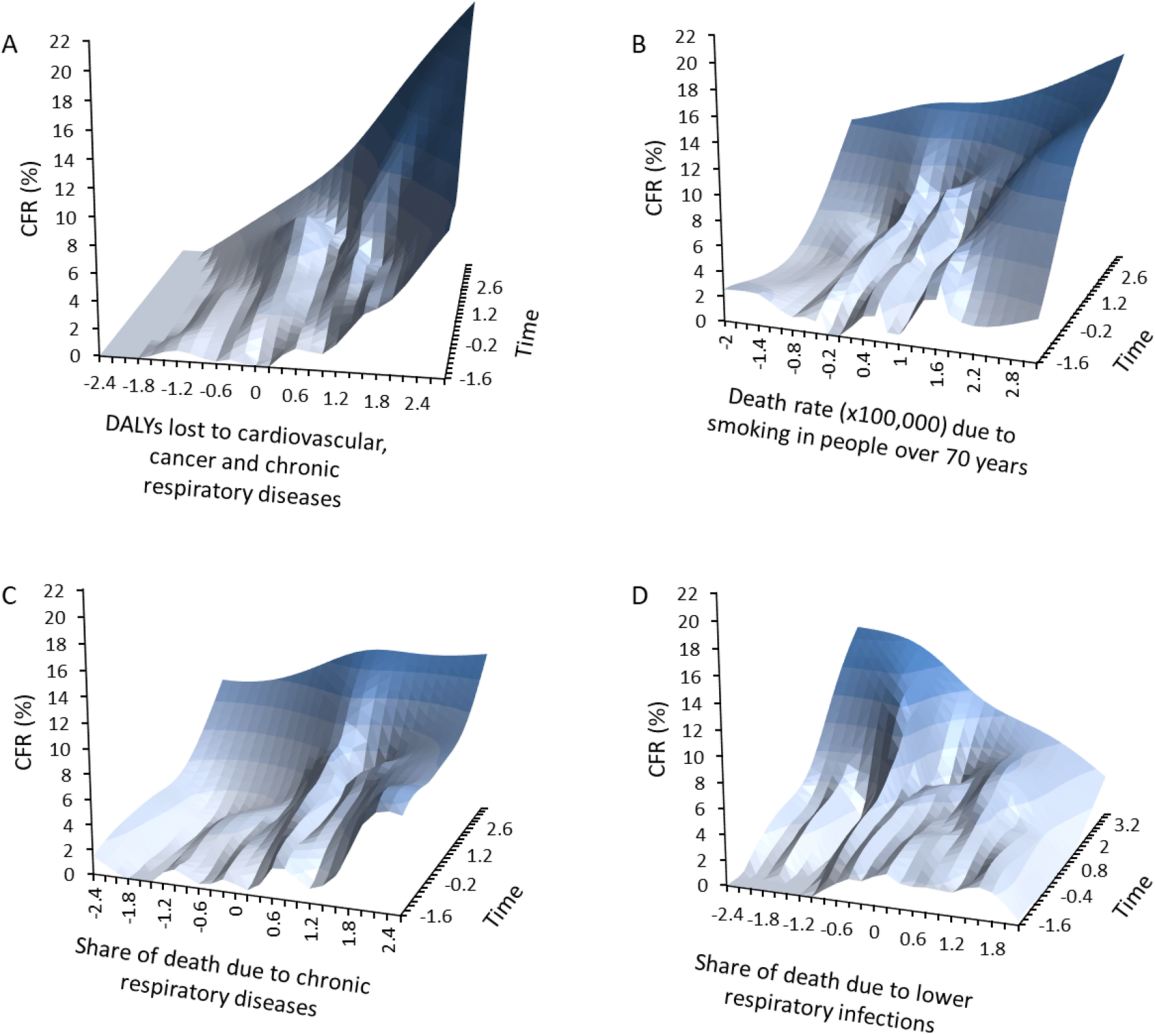
Time-dependent variation in COVID-19 case fatality rate (CFR) according to comorbidity factors. Time refers to the number of days between the date of occurrence of 1 death ×1,000,000 and June 11th 2020. (**A**) DALYs lost to cardiovascular, cancer and chronic respiratory diseases; (**B**) death rate (×100,000) due to smoking in people over 70 years; (**C**) share of death due to chronic respiratory diseases; (**D**) share of death due to lower respiratory infections. The surfaces were generated using a smoothed spline interpolation on the predicted values of the LMMs described in the text. Darker colors indicated higher values of CFR. X- and Y-axis are standardized values, allowing to have similarly scaled axis.

In order to uncover the factors that might account for such among-country variation in CFR, we ran four LMMs that included different fixed factors related to comorbidities, demographic, economic and political variables. These models provided evidence for several associations between country-specific risk factors and the temporal dynamics of CFR (Table 2). In particular, countries with high values of DALYs lost to cardiovascular, cancer and chronic respiratory diseases had high CFR (model 1, Table 2, Fig. 3A). Countries with high share of disease burden due to chronic respiratory diseases had high CFR (model 2, Table 2). Countries with the lowest death rate due to cardiovascular diseases had the lowest CFR and countries with the highest death rate due to smoking in people over 70 year-old had the highest CFR (model 3, Table 2, Fig. 3B). Finally, model 4 showed that CFR was positively associated with high share of death due to chronic respiratory, cardiovascular, kidney diseases and outdoor air pollution (Table 2, Fig. 3C). Interestingly, this model also showed a negative correlation between CFR and share of death due to lower respiratory infections (Table 2, Fig. 3D).

**Table 2:** Linear mixed models investigating the association between COVID-19 case fatality rate (CFR) and several descriptors of comorbidities, demographics, economics and political regime for each country.

Fixed effects	Estimate	SE	t	*p*	95% CI
**Model 1 (DALYs)**					
Intercept	1.243	2.000			
Time	− 1.607	0.257	− 6.26	** < 0.0001**	− 2.110/ − 1.104
Time^2^	1.267	0.261	4.85	** < 0.0001**	0.754/1.779
DALYs lost to cardiovascular, cancer and chronic respiratory diseases	− 0.569	5.381	− 0.11	0.9161	− 11.344/10.206
DALYs lost ×100,000 for people older than 70 year-old	0.161	0.943	0.17	0.8647	− 1.728/2.051
Time × DALYs lost to cardiovascular, cancer and chronic respiratory diseases	1.691	0.876	1.93	0.0537	− 0.027/3.408
Time × DALYs lost ×100,000 for people older than 70 year-old	0.164	0.190	0.87	0.3866	− 0.208/0.537
Time^2^ × DALYs lost to cardiovascular, cancer and chronic respiratory diseases	− 3.022	1.002	− 3.02	**0.0026**	− 4.986/ − 1.058
Time^2^ × DALYs lost ×100,000 for people older than 70 year-old	0.169	0.228	0.74	0.4604	− 0.279/0.617
*Covariance parameters*	Estimate	SE	z	*p*	
Variance	7.212	1.363	5.29	** < 0.0001**	
First-order autoregression	− 0.107	0.146	− 0.73	0.4633	
Residual	0.756	0.018	41.86	** < 0.0001**	
**Model 2 (share of disease burden)**					
Intercept	1.498	1.417			
Time	− 2.014	0.159	− 12.71	** < 0.0001**	− 2.324/ − 1.703
Time^2^	1.861	0.142	13.07	** < 0.0001**	1.582/2.141
Cardiovascular	0.080	0.836	0.10	0.9242	− 1.597/1.757
Cancer	2.209	1.327	1.66	0.1017	0.451/4.869
Chronic respiratory	0.314	0.602	0.52	0.6037	− 0.892/1.520
Time × cardiovascular	− 0.158	0.100	− 1.58	0.1151	− 0.354/0.039
Time × cancer	0.457	0.197	2.33	0.0201	0.072/0.843
Time × chronic respiratory	0.311	0.093	3.35	**0.0008**	0.129/0.493
Time^2^ × cardiovascular	0.183	0.110	1.67	0.0960	− 0.032/0.398
Time^2^ × cancer	0.048	0.215	0.22	0.8234	− 0.374/0.470
Time^2^ × chronic respiratory	− 0.630	0.098	− 6.43	** < 0.0001**	− 0.822/ − 0.438
*Covariance parameters*	Estimate	SE	z	*p*	
Variance	6.978	1.330	5.25	** < 0.0001**	
First-order autoregression	− 0.104	0.148	− 0.70	0.4835	
Residual	0.738	0.018	41.84	** < 0.0001**	
**Model 3 (age-standardized death rate)**					
Intercept	1.192	1.250			
Time	− 1.561	0.171	− 9.13	** < 0.0001**	− 1.896/ − 1.226
Time^2^	1.665	0.158	10.57	** < 0.0001**	1.356/1.974
Cardiovascular	− 0.941	0.784	− 1.20	0.2357	− 2.513/0.632
Cancer	0.441	0.616	0.72	0.4775	− 0.796/1.677
Air pollution	− 0.866	1.842	− 0.47	0.6402	− 4.559/2.827
Ambient particulate matter pollution	0.178	1.252	0.14	0.8877	− 2.333/2.689
Smoking in people older than 70 years	1.446	0.616	2.35	0.0226	0.211/2.681
Time × cardiovascular	− 0.349	0.108	− 3.24	**0.0012**	− 0.560/ − 0.138
Time × cancer	0.120	0.099	1.22	0.2244	− 0.074/0.315
Time × air pollution	0.611	0.363	1.69	0.0920	− 0.100/1.323
Time × ambient particulate matter pollution	0.089	0.253	0.35	0.7252	− 0.407/0.585
Time × smoking in people older than 70 years	0.730	0.115	6.35	** < 0.0001**	0.505/0.955
Time^2^ × cardiovascular	0.206	0.116	1.77	0.0769	− 0.022/0.434
Time^2^ × cancer	0.055	0.114	0.48	0.6299	− 0.169/0.279
Time^2^ × air pollution	− 0.285	0.439	− 0.65	0.5164	− 1.145/0.575
Time^2^ × ambient particulate matter pollution	− 0.125	0.316	− 0.40	0.6915	− 0.744/0.494
Time^2^ × smoking in people older than 70 years	− 0.860	0.127	− 6.77	** < 0.0001**	− 1.110/ − 0.611
*Covariance parameters*	Estimate	SE	z	*p*	
Variance	6.181	1.197	5.16	** < 0.0001**	
First-order autoregression	− 0.198	0.150	− 1.32	0.1877	
Residual	0.732	0.018	41.82	** < 0.0001**	
**Model 4 (share of death)**					
Intercept	1.533	1.314			
Time	− 1.994	0.168	− 11.88	** < 0.0001**	− 2.324/ − 1.665
Time^2^	2.017	0.153	13.22	** < 0.0001**	1.718/2.316
Cardiovascular	0.499	1.078	0.46	0.6456	− 1.667/2.664
Cancer	1.366	1.139	1.20	0.2357	− 0.920/3.652
Chronic respiratory	0.348	0.648	0.54	0.5937	− 0.953/1.648
Lower respiratory infections	− 0.085	0.596	− 0.14	0.8869	− 1.285/1.115
Kidney	0.263	0.723	0.36	0.7182	− 1.190/1.715
Diabetes mellitus	1.169	0.648	1.80	0.0771	− 0.132/2.469
Outdoor air pollution	− 0.236	0.765	− 0.31	0.7589	− 1.771/1.299
Time × cardiovascular	− 0.847	0.157	− 5.41	** < 0.0001**	− 1.153/ − 0.540
Time × cancer	− 0.402	0.171	− 2.34	0.0192	− 0.738/ − 0.065
Time × chronic respiratory	0.720	0.106	6.81	** < 0.0001**	0.512/0.927
Time × lower respiratory infections	− 0.601	0.101	− 5.94	** < 0.0001**	− 0.800/ − 0.403
Time × kidney	− 0.403	0.100	− 4.04	** < 0.0001**	− 0.599/ − 0.208
Time × diabetes mellitus	− 0.098	0.118	− 0.83	0.4066	− 0.330/0.134
Time × outdoor air pollution	0.374	0.111	3.35	**0.0008**	0.155/0.592
Time^2^ × cardiovascular	0.241	0.183	1.32	0.1870	− 0.117/0.600
Time^2^ × cancer	0.294	0.197	1.49	0.1355	− 0.092/0.679
Time^2^ × chronic respiratory	− 0.919	0.121	− 7.59	** < 0.0001**	− 1.156/ − 0.681
Time^2^ × lower respiratory infections	0.332	0.117	2.84	**0.0045**	0.103/0.561
Time^2^ × kidney	0.333	0.113	2.96	**0.0031**	0.112/0.554
Time^2^ × diabetes mellitus	− 0.068	0.134	− 0.50	0.6137	− 0.331/0.195
Time^2^ × outdoor air pollution	− 0.221	0.132	− 1.68	0.0937	− 0.479/0.037
*Covariance parameters*	Estimate	SE	z	*p*	
Variance	6.878	1.358	5.07	** < 0.0001**	
First-order autoregression	− 0.206	0.145	− 1.43	0.1535	
Residual	0.711	0.017	41.80	** < 0.0001**	

Each model included the same demographic, economic and political regime variables (GDP per capita, population size, total health care expenditure as share of GDP, number of hospital beds ×1000 inhabitants, share of the population over 70 years, political regime, stringency index and number of tests performed ×1000). In addition, model 1 included DALYs lost to cardiovascular, cancer and chronic respiratory diseases, and DALYs lost ×100,000 for people older than 70 years. Model 2 included share of disease burden (cardiovascular, cancer and chronic respiratory diseases). Model 3 included age-standardized death rates ×100,000 due to cardiovascular diseases, cancer, air pollution, ambient particulate air pollution, and smoking over 70 years. Model 4 included share of deaths for cardiovascular diseases, cancer, chronic respiratory diseases, lower respiratory diseases, diabetes, and outdoor air pollution. All models included time since number of deaths ×1,000,000 higher than 1 and squared time. Three nested factors were also included as random factors (continent, region within continent and country within region within continent).The table reports parameter estimates (with SE and 95% CI), t and p values (in bold significant p-values at the 0.01 threshold) for the comorbidity factors. Sample size is five continents, 17 geographical regions, 67 countries and 3596 observations.

**Figure 4:**
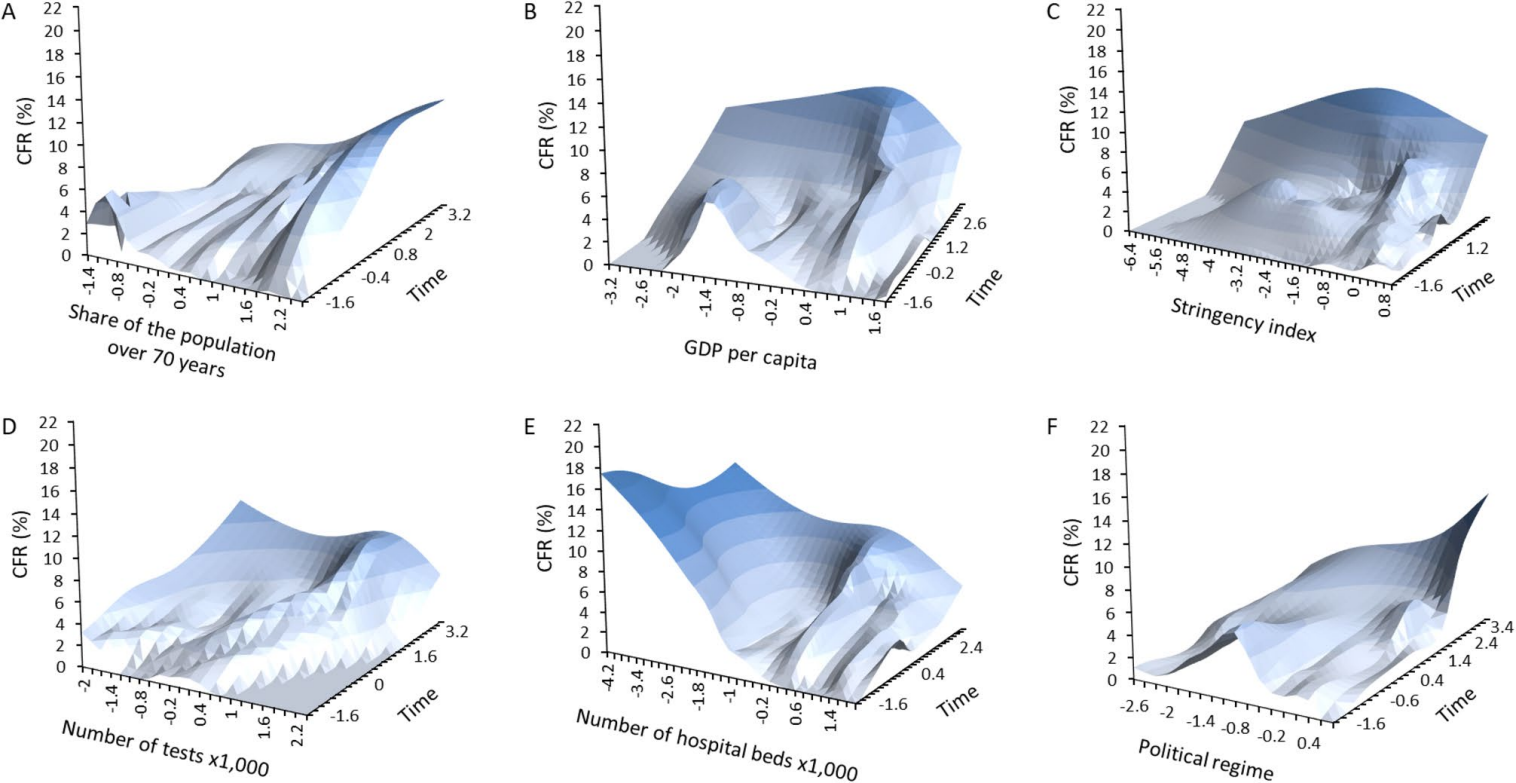
Time-dependent variation in COVID-19 case fatality rate (CFR) according to socio-economic factors. Time refers to the number of days between the date of occurrence of 1 death ×1,000,000 and June 11th 2020. (**A**) share of the population over 70 years; (**B**) GDP per capita; (**C**) stringency index; (**D**) number of tests ×1000; (**E**) number of hospital beds ×1000; (**F**) political regime. The surfaces were generated using a smoothed spline interpolation on the predicted values of the LMMs described in the text. Darker colors indicated higher values of CFR. X- and Y-axis are standardized values, allowing to have similarly scaled axis.

Among the demographic, economic and political variables, the four models consistently provided evidence for positive associations between the temporal dynamic of CFR and population size, GDP per capita, total health expenditure as share of GDP, share of the population over 70 years, stringency index (Table 3, Fig. 4A–C). As mentioned above, number of tests performed ×1000 showed a more complex pattern of association with CFR (Table 3, Fig. 4D). Two models (out of four) provided evidence for a negative association between number of hospital beds ×1000 and CFR (Table 3, Fig. 4E), while only one model showed a positive association between CFR and political regime, with democracies having the highest CFR (Table 3, Fig. 4F).

**Table 3:** Linear mixed models investigating the association between COVID-19 case fatality rate (CFR) and several descriptors of comorbidities, demographics, economics and political regime for each country.

	Model 1		Model 2		Model 3		Model 4	
	Estimate (SE)	*p*	Estimate (SE)	*p*	Estimate (SE)	*p*	Estimate (SE)	*p*
GDP per capita	0.427 (0.994)	0.6689	− 0.525 (1.003)	0.6028	− 0.090 (1.114)	0.9361	− 0.712 (1.050)	0.5010
Population size	3.308 (6.469)	0.6110	2.840 (0.807)	**0.0009**	2.815 (0.726)	**0.0003**	3.039 (0.894)	**0.0013**
Total health care expenditure as share of GDP	0.155 (0.602)	0.7977	0.089 (0.592)	0.8809	− 0.201 (0.609)	0.7432	− 0.138 (0.605)	0.8203
Number of hospital beds ×1000	− 1.592 (0.913)	0.0867	− 1.525 (0.942)	0.1111	− 1.712 (0.821)	0.0417	− 0.978 (0.924)	0.2950
Share of the population over 70 years	1.709 (1.274)	0.1852	0.421 (1.208)	0.7287	1.330 (1.008)	0.1929	1.465 (1.113)	0.1938
Political regime	− 0.183 (0.599)	0.761	− 0.952 (0.699)	0.1786	− 1.069 (0.735)	0.1518	− 0.989 (0.778)	0.2091
Stringency index	0.137 (0.047)	**0.0033**	0.127 (0.046)	**0.0057**	0.143 (0.046)	**0.0019**	0.111 (0.045)	0.0145
Number of tests performed ×1000	0.894 (0.119)	** < 0.0001**	1.001 (0.116)	** < 0.0001**	0.628 (0.123)	** < 0.0001**	0.948 (0.118)	** < 0.0001**
Time × GDP per capita	2.768 (0.168)	** < 0.0001**	1.874 (0.174)	** < 0.0001**	2.518 (0.200)	** < 0.0001**	2.111 (0.178)	** < 0.0001**
Time × population size	− 0.969 (1.046)	0.3541	1.030 (0.120)	** < 0.0001**	0.851 (0.121)	** < 0.0001**	0.466 (0.132)	**0.0004**
Time × total health care expenditure as share of GDP	0.658 (0.101)	** < 0.0001**	0.322 (0.097)	**0.0009**	0.753 (0.112)	** < 0.0001**	0.331 (0.098)	**0.0007**
Time × number of hospital beds ×1000	− 0.268 (0.101)	**0.0082**	− 0.148 (0.099)	0.1351	− 0.252 (0.090)	**0.0054**	0.265 (0.105)	0.0116
Time × share of the population over 70 years	0.451 (0.173)	**0.0091**	0.706 (0.162)	** < 0.0001**	0.888 (0.125)	** < 0.0001**	0.959 (0.143)	** < 0.0001**
Time × political regime	0.164 (0.102)	0.1072	− 0.117 (0.110)	0.2904	0.009 (0.133)	0.9487	0.214 (0.129)	0.0990
Time × stringency index	0.188 (0.073)	0.0106	0.107 (0.073)	0.1420	0.189 (0.075)	0.0115	0.342 (0.075)	** < 0.0001**
Time × number of tests performed ×1000	− 1.016 (0.096)	** < 0.0001**	− 1.038 (0.094)	** < 0.0001**	− 0.954 (0.097)	** < 0.0001**	− 1.106 (0.097)	** < 0.0001**
Time^2^ × GDP per capita	− 2.090 (0.190)	** < 0.0001**	− 1.135 (0.201)	** < 0.0001**	− 1.804 (0.229)	** < 0.0001**	− 1.589 (0.209)	** < 0.0001**
Time^2^ × population size	2.397 (1.198)	0.0454	− 1.131 (0.125)	** < 0.0001**	− 0.985 (0.129)	** < 0.0001**	− 0.0646 (0.136)	** < 0.0001**
Time^2^ × total health care expenditure as share of GDP	− 0.258 (0.105)	0.0146	0.079 (0.102)	0.4352	− 0.341 (0.116)	**0.0033**	− 0.037 (0.104)	0.7207
Time^2^ × number of hospital beds ×1000	0.398 (0.101)	** < 0.0001**	0.190 (0.096)	0.0479	0.273 (0.092)	**0.0030**	− 0.212 (0.109)	0.0513
Time^2^ × share of the population over 70 years	0.382 (0.188)	0.0429	− 0.368 (0.182)	0.0432	− 0.193 (0.131)	0.1399	− 0.300 (0.157)	0.0557
Time^2^ × political regime	− 0.328 (0.108)	**0.0024**	− 0.157 (0.117)	0.1787	− 0.264 (0.151)	0.0809	− 0.256 (0.151)	0.0901
Time^2^ × stringency index	− 0.026 (0.075)	0.7331	0.109 (0.075)	0.1481	− 0.040 (0.077)	0.6042	− 0.120 (0.077)	0.1233
Time2 × number of tests performed ×1000	0.340 (0.110)	**0.0020**	0.386 (0.108)	**0.0004**	0.342 (0.112)	**0.0023**	0.487 (0.114)	** < 0.0001**

Each model included the same demographic, economic and political regime variables (GDP per capita, population size, total health care expenditure as share of GDP, number of hospital beds ×1000 inhabitants, share of the population over 70 years, political regime, stringency index and number of tests performed ×1000). In addition, model 1 included DALYs lost to cardiovascular, cancer and chronic respiratory diseases, and DALYs lost ×100,000 for people older than 70 years. Model 2 included share of disease burden (cardiovascular, cancer and chronic respiratory diseases). Model 3 included age-standardized death rates ×100,000 due to cardiovascular diseases, cancer, air pollution, ambient particulate air pollution, and smoking over 70 years. Model 4 included share of deaths for cardiovascular diseases, cancer, chronic respiratory diseases, lower respiratory diseases, diabetes, and outdoor air pollution. All models included time since number of deaths ×1,000,000 higher than 1 and squared time. Three nested factors were also included as random factors (continent, region within continent and country within region within continent). The table reports parameter estimates (SE), and p values for socio-economic factors in each model (in bold significant p-values at the 0.01 threshold). Sample size is five continents, 17 geographical regions, 67 countries and 3596 observations.

## Discussion

As the SARS-CoV-2 epidemic continues to spread worldwide, the mortality induced by the disease has been a serious matter of concern, with some countries paying a high toll to the infection. In this light, it is important to understand why some countries seem to experience lower mortality rate than others and possibly to uncover the associated factors. Here, we showed that CFR greatly differs among countries (even after controlling for the number of tests and the severity of social distancing rules), and several comorbidity (DALYs lost to cardiovascular, cancer and chronic respiratory diseases; death rate due to smoking in people older than 70; share of deaths due to cardiovascular, chronic respiratory, kidney diseases), and socio-economic factors (population size, GDP per capita, share of the population over 70, number of hospital beds ×1000, political regime) were positively associated with COVID-19 CFR.

Assessing case fatality rate during an ongoing outbreak is particularly difficult for, at least two reasons^11,19^. CFR at a given time might be an underestimate of the final mortality rate because cumulative number of deaths will eventually keep increasing as some patients are still hosted in intensive care units (i.e., right censoring). Conversely, CFR might overestimate actual mortality if a large fraction of infected people do not develop disease symptoms (or develop mild symptoms), get unnoticed and are not included into the population of confirmed cases. Several countries have implemented a strategy of massive screening, aiming at identifying positives and isolating them, as to avoid further spreading of the disease. Data on the number of tests performed per 1000 people, therefore, allow investigating whether countries with a massive screening policy were also those with the lowest CFR. The results showed no simple association between CFR and testing, with countries showing the expected negative relationship, while others showing a positive correlation. This suggests that differences in population screening are not enough to explain the tremendous variation in CFR among countries. As mentioned above, the other possible bias when computing CFR is that some of the patients who are still in intensive care units may eventually die, and therefore if the total number of confirmed cases remains unchanged, CFR might still increase. However, visual inspection of the temporal trajectories of CFR shows that values have reached an asymptote for the vast majority of countries (Fig. 1). This suggests that, unless a second wave hits in the following weeks/months, we should not expect CFR to substantially vary as a consequence of residual mortality.

Despite the uncertainty associated with the estimation of CFR, its comparison between countries can provide useful insights into the heterogeneity in the burden paid to the disease. We showed that the trajectories of time dependent variation in CFR greatly differed among countries, while controlling for differences in testing strategies and stringency index. This quantitatively corroborates and statistically validates the intuition that some countries better dealt with the disease than others. The following step was to try to understand whether such heterogeneity arises as the consequence of predictable factors. Previous reports on the clinical outcome of the disease have identified two major factors associated with poor prognosis: age and the presence of comorbidities. Elderly people have been shown to suffer the highest mortality rate following infection with SARS-CoV-2^11,19^. Similarly, previous history of cardiovascular disorders, cancer and diabetes have been reported to substantially increase COVID-19 mortality risk^20,21^. We therefore predicted that countries with a higher share of elderly people and a higher incidence of known comorbidity factors might suffer from the highest CFR.

Our integrated modelling approach provided some evidence in support to these predictions. In particular, several metrics of comorbidity factors were positively associated with the temporal dynamics of CFR (DALYs lost to cardiovascular, cancer and chronic respiratory diseases; share of burden due to chronic respiratory diseases; death rate due to cardiovascular diseases; death rate due to smoking in people older than 70; share of death due to chronic respiratory and kidney diseases). Interestingly, our model also showed that share of death due to lower respiratory infections was negatively associated with COVID-19 CFR. This negative association is intriguing, especially in the light of recent reports of possible cross-immunity that might confer partial protection to SARS-CoV-2^22^.

All the above-mentioned associations between comorbidities and CFR hold while controlling for several potential confounding factors describing the socio-economic context of different countries. As such, positive associations between comorbidities and CFR at the country level, do not merely reflect differences in the structure of the age-pyramid, or the amount of resources allocated to the health care system. Actually, focusing on such demographic and socio-economic factors allowed us to identify several other variables that contributed to explain among-country variation in CFR. As predicted, countries with the highest share of elderly people (over 70) also had the highest CFR.

Economic parameters might equally well contribute to shape COVID-19 mortality. As the number of severe cases increases during the epidemic, the health care system can get overwhelmed and might be unable to receive and treat all those who need intensive care. Mortality might therefore results from health care systems that are inadequate to deal with large number of cases requiring simultaneous admittance in intensive care units. We used several proxies describing the investment of each country into the health care system and found a negative association between the number of hospital beds per 1000 inhabitants and CFR. However, seemingly in contradiction to this view, we also found that CFR was highest in countries with high GDP per capita and high total health expenditure as share of GDP. While odd, this result corroborates the impression that wealthy countries in Europe and North America have paid a severe toll to the infection. Overall, the relationship between investment into health care system and CFR appear to be more complex than one might expect.

A final source of variation accounting for differences in CFR might be due to differential reports of number of deaths and/or confirmed cases between countries. This might reflect different counting/reporting methodology (e.g., testing strategy, deciding whether or not a given patient died because of COVID-19). In addition, most of the countries have been implementing social distancing protocols that differed in the severity of the restrictions imposed and on the timing of policy execution. Moreover, different populations might follow governmental instructions more or less loosely, due to the perceived risk/benefit of applying such instructions. For instance, social distancing and isolation might be more easily applied in countries with autocratic regimes that exert a more stringent control over the population. The role of social and cultural traits in the emergence of zoonotic diseases has already been discussed in the past, including the idea that collectivistic societies might have built as a way to better control epidemic waves^23,24^. We explored how political regime and the severity of isolation policies were associated with CFR. We found moderate evidence (1 out of 4 models) suggesting that countries with a democratic regime were those with the highest CFR. The analysis of the stringency index, describing the severity of the restrictions implemented by each country, showed that highest values of CFR were reached for intermediate values of the stringency index. This might reflect different processes. First, countries where the epidemic wave was relatively low (perhaps because of the factors described above) could have implemented relatively mild restriction policies, compared to countries where the epidemic got out of control and that required imposing more stringent social distancing rules.

Although we report here some associations between comorbidities, demographic, socio-economic variables and COVID-19 CFR, we fully acknowledge that these factors do not perfectly explain the variation in CFR among countries. This might come from the coarse grain of the analyses (country level), the error associated with the metrics used in our study, the role played by other factors not taken into account in our study, or the uncertainties associated with the estimation of CFR while the epidemic is still ongoing. If serological tests will be used on a very large scale to assess the proportion of the population that has been infected by the virus, we will have a better estimate of the mortality rate and the possible factors explaining the among-country heterogeneity. With this in mind, we nevertheless believe that our results stress the role of comorbidities, socio-economic and political factors as potential drivers affecting how a country deals with globally threatening epidemics.

## Supplementary information

Supplementary Table S1.

## Data Availability

The full dataset is available in the online appendix.
