# Supplementary material for: Explaining among-country variation in COVID-19 case fatality rate
Source: Sci Rep. 2020 Nov 3;10:18909. doi: 10.1038/s41598-020-75848-2 (PMC7609641; doi:10.1038/s41598-020-75848-2)
Supplement: Supplementary file 1 — Supplementary Table S1. [file 41598_2020_75848_MOESM1_ESM.pdf]

Supplementary material

Why does COVID-19 case fatality rate vary among countries?

Gabriele Sorci[1*], Bruno Faivre[1], Serge Morand[2,3]

1. Biogéosciences, CNRS UMR 6282, Université de Bourgogne France-Comté, Dijon, France
2. CNRS ISEM - CIRAD ASTRE - Montpellier Université, Montpellier, France
3. Faculty of Tropical Medicine, Mahidol University, Bangkok, Thailand

*Corresponding author:

Gabriele Sorci, Biogéosciences, CNRS UMR 6282, Université de Bourgogne Franche-Comté, 6 Boulevard Gabriel, 21000 Dijon, France

Email : gabriele.sorci@u-bourgogne.fr

Table S1. List of variable used. All data were retrieved from the online resource https://ourworldindata.org/.

Variable	Type	Source
Continent	class	
Geographical region	class	United Nations
Country	class	European Center for Disease Prevention and Control
Time since 100th SARS-CoV-2 case (up to June 11th 2020)	continuous	European Center for Disease Prevention and Control
Time since the occurrence of 1 death x1,000,000 (up to June 11th 2020)	continuous	European Center for Disease Prevention and Control
COVID-19 case fatality rate (up to June 11th 2020)<br>Sex: both<br>Age: all ages	continuous	European Center for Disease Prevention and Control
Disability-adjusted life years (numbers) Cardiovascular diseases<br>Sex: both<br>Age: all ages	continuous	Global Burden of Disease Collaborative Network. Global Burden of Disease Study 2017 (GBD 2017) Results. Seattle, United States: Institute for Health Metrics and Evaluation (IHME), 2018.
Disability-adjusted life years (numbers) Cancers<br>Sex: both<br>Age: all ages	continuous	Global Burden of Disease Collaborative Network. Global Burden of Disease Study 2017 (GBD 2017) Results. Seattle, United States: Institute for Health Metrics and Evaluation (IHME), 2018.
Disability-adjusted life years (numbers) Chronic respiratory diseases<br>Sex: both<br>Age: all ages	continuous	Global Burden of Disease Collaborative Network. Global Burden of Disease Study 2017 (GBD 2017) Results. Seattle, United States: Institute for Health Metrics and Evaluation (IHME), 2018.
Share of total disease burden (%)<br>Cardiovascular diseases<br>Sex: both<br>Age: all ages	continuous	Global Burden of Disease Collaborative Network. Global Burden of Disease Study 2016 (GBD 2016) Results. Seattle, United States: Institute for Health Metrics and Evaluation (IHME), 2017.
Share of total disease burden (%)<br>Cancers<br>Sex: both<br>Age: all ages	continuous	Global Burden of Disease Collaborative Network. Global Burden of Disease Study 2016 (GBD 2016) Results. Seattle, United States: Institute for Health Metrics and Evaluation (IHME), 2017.
Share of total disease burden (%)<br>Chronic respiratory diseases<br>Sex: both<br>Age: all ages	continuous	Global Burden of Disease Collaborative Network. Global Burden of Disease Study 2016 (GBD 2016) Results. Seattle, United States: Institute for Health Metrics and Evaluation (IHME), 2017.
Disability-adjusted life years (rate)<br>All causes<br>Sex: both	continuous	Global Burden of Disease Collaborative Network. Global Burden of Disease Study 2017 (GBD 2017) Results. Seattle, United States: Institute for Health Metrics and Evaluation (IHME), 2018.

Age: 70+ years		
Deaths (rate)<br>Cardiovascular diseases<br>Sex: both<br>Age: age-standardized	continuous	Global Burden of Disease Collaborative Network. Global Burden of Disease Study 2017 (GBD 2017) Results. Seattle, United States: Institute for Health Metrics and Evaluation (IHME), 2018.
Deaths (rate)<br>Cancers<br>Sex: both<br>Age: age-standardized	continuous	Global Burden of Disease Collaborative Network. Global Burden of Disease Study 2017 (GBD 2017) Results. Seattle, United States: Institute for Health Metrics and Evaluation (IHME), 2018.
Deaths (rate)<br>Air pollution<br>Sex: both<br>Age: age-standardized	continuous	Global Burden of Disease Collaborative Network. Global Burden of Disease Study 2017 (GBD 2017) Results. Seattle, United States: Institute for Health Metrics and Evaluation (IHME), 2018.
Deaths (rate)<br>Ambient particulate matter pollution<br>Sex: both<br>Age: age-standardized	continuous	Global Burden of Disease Collaborative Network. Global Burden of Disease Study 2017 (GBD 2017) Results. Seattle, United States: Institute for Health Metrics and Evaluation (IHME), 2018.
Deaths (rate)<br>Smoking<br>Sex: both<br>Age: 70+ years	continuous	Global Burden of Disease Collaborative Network. Global Burden of Disease Study 2017 (GBD 2017) Results. Seattle, United States: Institute for Health Metrics and Evaluation (IHME), 2018.
Share of deaths (%)<br>Cardiovascular diseases<br>Sex: both<br>Age: all ages	continuous	Global Burden of Disease Collaborative Network. Global Burden of Disease Study 2017 (GBD 2017) Results. Seattle, United States: Institute for Health Metrics and Evaluation (IHME), 2018.
Share of deaths (%)<br>Cancers<br>Sex: both<br>Age: all ages	continuous	Global Burden of Disease Collaborative Network. Global Burden of Disease Study 2017 (GBD 2017) Results. Seattle, United States: Institute for Health Metrics and Evaluation (IHME), 2018.
Share of deaths (%)<br>Chronic respiratory diseases<br>Sex: both<br>Age: all ages	continuous	Global Burden of Disease Collaborative Network. Global Burden of Disease Study 2017 (GBD 2017) Results. Seattle, United States: Institute for Health Metrics and Evaluation (IHME), 2018.
Share of deaths (%)<br>Chronic kidney diseases<br>Sex: both<br>Age: all ages	continuous	Global Burden of Disease Collaborative Network. Global Burden of Disease Study 2017 (GBD 2017) Results. Seattle, United States: Institute for Health Metrics and Evaluation (IHME), 2018.
Share of deaths (%)<br>Lower respiratory infections<br>Sex: both<br>Age: all ages	continuous	Global Burden of Disease Collaborative Network. Global Burden of Disease Study 2017 (GBD 2017) Results. Seattle, United States: Institute for Health Metrics and Evaluation (IHME), 2018.
Share of deaths (%)<br>Diabetes mellitus<br>Sex: both<br>Age: all ages	continuous	Global Burden of Disease Collaborative Network. Global Burden of Disease Study 2017 (GBD 2017) Results. Seattle, United States: Institute for Health Metrics and Evaluation (IHME), 2018.
Share of premature deaths attributed to outdoor air pollution (ozone and particulate matter) (%)<br>Sex: both	continuous	Global Burden of Disease Collaborative Network. Global Burden of Disease Study 2017 (GBD 2017) Results. Seattle, United States: Institute for Health Metrics and Evaluation (IHME), 2018.

Age: all ages		
Gross domestic product per capita based on purchasing power parity	continuous	World Bank – World Development Indicators
Total population	continuous	Gapminder, HYDE (2016) and United Nations Population Division (2019)
Total health expenditure as share of GDP (%)	continuous	World Bank – World Development Indicators
Number of hospital beds	continuous	OECD; Eurostat; World Bank; National Government Records as reported in https://ourworldindata.org/.
Share of the population aged 70 or over	continuous	United Nations, Department of Economic and Social Affairs, Population Division (2017). World Population Prospects: The 2017 Revision, DVD Edition.
Level of democracy (from -10 to 10)	continuous	Polity IV as reported in https://ourworldindata.org/.
Number of tests performed x 1,000	continuous	https://ourworldindata.org/.
Stringency index	continuous	https://ourworldindata.org/.
